# Redefining health priorities: Sub-Saharan Africa’s new frontier in disease burden

**DOI:** 10.7189/jogh.14.03049

**Published:** 2024-12-20

**Authors:** Taddese A Zerfu, Amare A Tareke, Sibhatu Biadgilign

**Affiliations:** 1 International Food Policy Research Institute (IFPRI), Addis Ababa, Ethiopia; 2 College of Medicine and Health Sciences, Wollo University, Dessie, Ethiopia; 3 Independent Public Health Analyst and Research Consultant, Addis Ababa, Ethiopia

Recent Lancet Global Burden of Disease Study projections highlight a significant transformation in sub-Saharan Africa’s (SSA) health landscape by 2050 [1]. Although global age-standardised disease burden and life expectancy are anticipated to improve, SSA will move from primarily impacted by communicable, maternal, neonatal, and nutritional diseases (CMNNs) to an increasing prevalence of non-communicable diseases (NCDs). This evolving scenario requires urgent modifications in public health strategies to tackle both ongoing and emerging health challenges.

Historically, SSA has grappled with CMNNs [2–4], but an increasing incidence of NCDs, such as cardiovascular diseases, diabetes, and cancers, is becoming evident [4,5]. This rise is fuelled by demographic changes, urbanisation, and evolving lifestyle factors [5–7]. The health care systems, primarily focused on CMNNs [7–9], now face significant challenges in addressing this dual burden. To manage these conditions effectively, substantial investments in health care infrastructure, expanded diagnostic and treatment facilities, and enhanced training for health care professionals are essential.

Developing a robust health care system that can manage both CMNNs and NCDs is crucial for improving access to care and overall health outcomes. This requires strategic adjustments in health policies and practices to address current and emerging health challenges. Ethiopia could be a good example to illustrate how missed opportunities in health policy adjustments can impact the management of both CMNNs and NCDs. The country’s Health Extension Program (HEP) trains community health workers to provide essential services, enhancing health care infrastructure and promoting preventive care. However, the program has emphasised CMNNs over NCDs. By building on the existing HEP, Ethiopia could better manage the dual burden of disease and improve overall health outcomes. By recalibrating health strategies, countries in SSA can better respond to this evolving disease burden.

Addressing NCDs demands a comprehensive approach that goes beyond traditional health care practices. Strengthening health care systems to provide quality services and ensure universal access to affordable care is vital. In Ethiopia, this can be seen through the HEP, which not only enhances health care infrastructure but also empowers community health workers to deliver essential services. This program exemplifies how structured frameworks for community engagement and collaboration with local organisations can effectively address health challenges. Outreach programs emphasising culturally relevant education have shown promise in increasing awareness of NCDs. Training community health workers to deliver effective NCD prevention and management education is also critical. Policymakers can effectively address this by developing standardised programs focused on NCD prevention. Collaborating with health care organisations ensures comprehensive, evidence-based training, while ongoing professional development keeps community health workers updated on best practices and emerging strategies.

Policymakers should leverage local health data to inform decisions, prioritise budgets for NCD initiatives, and foster partnerships that create a holistic health approach. Such strategies can enhance community involvement and resource efficiency, ultimately improving health outcomes across the region. Ethiopia’s experience highlights the potential for integrated approaches to tackle both CMNNs and NCDs, paving the way for more resilient health care systems in sub-Saharan Africa.

In addition to long-term prevention efforts, addressing acute management of NCDs, like cardiac events and strokes, is essential. A balanced approach that incorporates both preventive strategies and immediate life-saving interventions is crucial for managing the challenges posed by NCDs. Integrating acute care strategies within the broader health framework can facilitate timely responses that complement ongoing prevention efforts.

Implementing pilot programs with measurable goals, such as increasing access to care for both CMNNs and NCDs by 15% within two years, could be a practical approach. Prioritising public health advocacy campaigns that promote healthy lifestyles, such as dietary diversity, physical activity, and the reduction of alcohol and tobacco use, will be essential. Supporting policies, such as tax incentives for healthy foods and regulations on tobacco products, can further enhance these efforts. Additionally, establishing measurable objectives, like achieving a 10% reduction in smoking rates over five years, could help track progress effectively.

Moreover, establishing early screening programs for high-risk populations can lead to earlier diagnoses and more effective management of chronic conditions. Effective policy frameworks, including risk reduction strategies and adequate resource allocation, are crucial for integrating NCD management into existing health care systems. Successful models from other regions, such as the Philippines’ Universal Health Care program [10], Brazil’s Mais Médicos initiative, and Thailand’s Healthy Thailand initiative, demonstrate the benefits of holistic approaches to improve health outcomes.

Community engagement is vital for fostering collaboration between governments, NGOs, and the private sector. Implementing targeted awareness campaigns can increase preventive health care visits and reduce preventable diseases, aiming to double visit frequency and achieve a 50% reduction in preventable disease cases over five years.

Addressing socio-economic determinants like poverty and education is essential for reducing health inequities. By integrating NCDs into national health strategies and leveraging innovation and technology, SSA can effectively navigate the transition from CMNNs to the rising burden of NCDs. A holistic approach emphasising prevention, policy development, and community engagement will be critical in mitigating the impact of NCDs and enhancing health outcomes.

To effectively expand diagnostic and treatment facilities, governments and health care institutions must prioritize the construction and upgrading of health care centres, particularly in underserved areas. Specifically, governments should aim to double the number of diagnostic centres within the next five years. Additionally, they should invest in training programs for health care professionals and allocate at least 15% of their health budgets to these initiatives. This targeted approach would significantly enhance access to essential health care services and improve overall health outcomes in these communities.

Addressing the rising burden of NCDs also requires leveraging technology and fostering innovation. SSA countries should prioritise major NCDs by adopting digital health consultations and mobile health applications for remote monitoring, thus extending services to underserved populations. Community-based screening programs and data analytics to identify high-risk groups can further enhance targeted interventions. Investing in training for health care workers in evidence-based practices and promoting research on local risk factors will support effective NCD management within primary health care systems.

Supporting innovation is vital. Encouraging the development of new diagnostic tools and therapeutic approaches can revolutionise the prevention, diagnosis, and treatment of NCDs. By embracing advancements, health care systems can deliver more effective care, improve outcomes, and reduce the overall burden of NCDs. To enhance health data quality and reduce reporting lags, we propose decentralized data collection hubs equipped with backup power, mobile data units, and cloud storage. By training personnel and forming partnerships with telecommunications providers, we aim for real-time monitoring, ensuring reliable data collection and timely reporting, even during power failures.

Lastly, to effectively address the critical bottlenecks and challenges posed by financial and infrastructural barriers, we recommend solutions, including the implementation of innovative financing mechanisms and increased health care budget allocations, which are essential for sustainable progress. In addition, strengthening supply chain management for essential medicines will ensure their availability and accessibility. Enhancing digital health infrastructure and improving transportation networks will foster a more resilient health system, ultimately leading to better health outcomes.

In conclusion, sub-Saharan Africa faces a pivotal shift in disease burden, transitioning from CMNNs to an increasing prevalence of NCDs. While global health metrics are set to improve, SSA countries must adopt a multidisciplinary, multi-sectoral strategy to address this transition. Strengthening health care systems, prioritising public health advocacy, integrating NCD management into national policies, and emphasising early screening and prevention will be crucial. By addressing socioeconomic determinants and fostering innovation, SSA can improve health outcomes and equity, ultimately contributing to sustainable development goals and a healthier future.
